# Regional variations in excessive polypharmacy and potentially inappropriate drug use among older adults in Sweden: Trends from 2006 to 2020

**DOI:** 10.3389/fphar.2023.1030849

**Published:** 2023-02-08

**Authors:** Jonas W. Wastesson, Johan Fritzell, Bo Burström, Kristina Johnell, Johan Fastbom

**Affiliations:** ^1^ Department of Medical Epidemiology and Biostatistics, Karolinska Institutet, Stockholm, Sweden; ^2^ Aging Research Center, Karolinska Institutet and Stockholm University, Stockholm, Sweden; ^3^ Department of Global Public Health, Karolinska Institutet, Stockholm, Sweden; ^4^ National Board of Health and Welfare (Socialstyrelsen), Stockholm, Sweden

**Keywords:** aged, ageing population, scoping, polypharmacy source: MeSH, inappropriate drug use, trends (source: MeSH NLM), regional variability

## Abstract

**Introduction:** Potentially inappropriate drug use (PID) is common among older adults. Cross-sectional data suggest that there are marked regional variations in PID in Sweden. There is, however, a lack of knowledge about how the regional variations have changed over time.

**Objectives:** This study aimed to investigate the regional differences in the prevalence of PID in Sweden, 2006–2020.

**Methods:** In this repeated cross-sectional study, we included all older adults (≥75 years) registered in Sweden, yearly from 2006 to 2020. We used nationwide data from the Swedish Prescribed Drug Register linked at the individual level to the Swedish Total Population Register. We selected three indicators of PID according to the Swedish national “Quality indicators for good drug therapy in the elderly”: 1) Excessive polypharmacy (use of ≥10 drugs); 2) Concurrent use of three or more psychotropic drugs; 3) Use of “drugs that should be avoided in older adults unless specific reasons exist.” The prevalence of these indicators was calculated for each of Sweden’s 21 regions, yearly from 2006 to 2020. The annual coefficient of variation (CV) was calculated for each indicator by dividing the standard deviation of the regions by the national average, to measure relative variability.

**Results:** In the population of about 800,000 older adults per year, the national prevalence of “drugs that should be avoided in older adults,” was reduced by 59% from 2006 to 2020. There was a slight decline in the use of three or more psychotropics, while the prevalence of excessive polypharmacy increased. The CV for excessive polypharmacy was 14% in 2006 and 9% in 2020 compared to 18% and 14% for “use of three or more psychotropics”, and stable at around 10% for ‘drugs that should be avoided in older adults.’

**Conclusions:** The regional variation in potentially inappropriate drug use decreased or were stable from 2006 to 2020. The regional differences were largest for the use of three or more psychotropics. We found a general tendency that regions with a good performance at the start of the period performed well across the entire period. Future studies should investigate the reasons for regional variation and explore strategies to reduce unwarranted differences.

## 1 Introduction

Potentially inappropriate drug use (PID) is common among older adults (Guaraldo et al., 2011; Opondo et al., 2012; Hill-Taylor et al., 2013; Tommelein et al., 2015). PID is associated with adverse drug events, hospitalisations and mortality (Muhlack et al., 2017; Xing et al., 2019). In Sweden, many indicators of PID and hazardous drug use have decreased over time (e.g., “Drugs that should be avoided in older adults unless specific reasons exist”, use of antipsychotic drugs, and potential drug-drug interactions) whereas some have been stable or even increased (e.g., excessive polypharmacy) (Hovstadius et al., 2013; Thorell et al., 2020). Large regional variations in the prevalence of PID have been reported for specific years for Sweden (Johnell et al., 2007; Socialstyrelsen, 2017a). However, the long-term trends in these regional differences have not been investigated.

PID among older adults is frequently assessed using consensus-based explicit criteria. Internationally, there exist a number of lists of inappropriate drugs for older adults, for example Beers criteria (Fick et al., 2012; Samuel, 2015; Fick et al., 2019) and STOPP/START criteria (Gallagher et al., 2008; O’mahony et al., 2015). In Sweden, the most frequently used are the “Indicators for good drug therapy in the elderly”, introduced by the Swedish National Board of Health and Welfare in 2004 (Socialstyrelsen, 2004) and continuously updated in 2010 (National Board of Health and Welfare, 2010) and 2017 (Socialstyrelsen, 2017b). The different sets of criteria typically share many features and include similar drugs, although some variations exists, partly due to differences in the national drug formularies (Morin et al., 2015). For a comparison between the previous versions of the Swedish criteria and other lists, see Morin et al, (2015) and Fastbom and Johnell, (2015). We selected three of the most general indicators from the Swedish criteria to examine regional variations over time.

Regional variations in drug use can occur for several reasons, often divided into contextual and individual/compositional factors (Morgan et al., 2010). Contextual factors are generally factors distal to the individual, describing the context in which medications are prescribed and consumed. In Sweden, the overall responsibility for medication policy belong to the 21 regions (Wettermark et al., 2008). Each region has its own medication committee making recommendations and governing the drug prescribing in their region. Thus, possible contextual factors may be related to the recommendations issued by the medication committee in each region. This is for example done by producing formulary of essential medicines, most notably the “Wise List” issued by Stockholm healthcare region (Eriksen et al., 2017). Another contextual factor may be “therapeutic traditions” (Ohlsson et al., 2009). This implies that prescribers sharing a common workplace or geographical proximity have similar prescribing patterns. Individual/compositional factors are instead about differences in population characteristics across regions, i.e., inhabitants of a certain region might be different in relation to age, sex, socioeconomics, and health status (Morgan et al., 2010).

Regional variations in general drug use and for specific classes are frequently reported in the literature (Wangia and Shireman, 2013). Fewer studies have investigated trends in regional differences in drug use for older adults (Hogan et al., 2003; Naughton et al., 2006; Jirón et al., 2016; Hyttinen et al., 2019; Nothelle et al., 2019). A notable exception is a Canadian study, finding persistent and unexplained regional variation in commonly used drugs by older adults (Hogan et al., 2003). The differences included both variation in the number of used drugs and type of drugs across the regions. The significant differences identified in that study did not match the regional differences in medical conditions or drug benefit plan. Hence, the authors concluded that the reasons for the regional variation were largely unexplained.

Understanding regional variations in trends of PID is important to describe prescribing patterns and identify regions where performance could be improved. Furthermore, describing regional trends can also serve to generate hypotheses about the causes of these differences. Therefore, this study aimed to i) investigate the overall trend of PID in Sweden 2006–2020, ii) to explore regional variations in this trend.

To this end, we have used data from the nationwide Swedish Prescribed Drug Register (SPDR) to analyse drug use in persons 75 years and older during the years 2006–2020, focusing on three indicators of PID from the Swedish criteria: excessive polypharmacy, use of three or more psychotropic drugs and use of “drugs that should be avoided in older adults unless specific reasons exist.”

## 2 Materials and methods

### 2.1 Data source

The current study was based on routinely collected data in Sweden, a country with a universal healthcare system. The data were extracted from two Swedish nationwide population-based registers, linked by the unique personal identity number, pseudonymised to the researchers: 1) The Total Population Register at Statistics Sweden provided information about who were residents in Sweden, as well as dates of deaths and moving in/out of the country during the study period (Ludvigsson et al., 2016). 2) The Swedish Prescribed Drug Register (SPDR) at the Swedish National Board of Health and Welfare provided information on all prescribed drugs purchased at pharmacies in Sweden (Wettermark et al., 2007).

### 2.2 Study design and population

This is a repeated cross-sectional study including all individuals aged 75 years and older and registered as living in Sweden, each year from 2006 to 2020.

### 2.3 Assessment of outcomes

Data on drug use were extracted from the SPDR. Current drug use on 31 December each year was calculated for each individual, based on the date of drug dispensing, the total amount of drug dispensed and the prescribed daily dose, as previously described (Wallerstedt et al., 2013). The number of different drugs used on the index date is presented as the number of distinct brand names according to the 5th level of Anatomical Therapeutic Chemical (ATC) classification system.

To assess the extent and quality of drug use in older persons, we operationalised three indicators from the Swedish national “Indicators for good drug therapy in the elderly” (Fastbom and Johnell, 2015):

Use of 10 or more drugs (definition of excessive polypharmacy), the number of distinct brand names according to the 5th level of the ATC classification system.

Use of three or more psychotropic drugs (i.e., belonging to ATC-groups N05A, N05B, N05C or N06A; Supplementary Table S1).

Drugs that “should be avoided in older adults unless specific reasons exist” (inappropriate drugs) (list of ATC codes available in Supplementary Table S2).

### 2.4 Statistical analysis

Descriptive statistics were used for illustrating the geographical distribution of the three indicators. In order to have a standardised measure of the regional variability, we calculated the annual coefficient of variation (CV), by dividing the standard deviation of the regions by the national average, for each indicator and year. Further, we calculated how the prevalence of each region diverged from the national average for each year and indicator, in order to display the relative difference between regions. As a supplementary analysis, we provide the ranking of the regions in year 2006 and 2020 for each indicator, to display the regions relative performance across the study period. As a *post hoc* analysis, we report the 10 most frequently used psychotropic drugs and “drugs that should be avoided in older adults unless specific reasons exist” in year 2006 and 2020. This was done in order to display changes in item composition over the period. The Statistical Package for the Social Sciences (SPSS Statistics, version XX, Chicago, IL) was used for the analyses.

### 2.5 Ethical approval

The study was approved by the Regional Ethical Review Board in Stockholm (2016/1001–31/4, 2020–03525; 2021–02004).

## 3 Results

More than 800,000 individuals aged 75 years and older were included each year from 2006 to 2020. The mean age was about 82 years each study year, and the proportion of females was 61% in 2006 and 56% in 2020 (Table 1). Nationally, there was a 7% increase in the mean number of drugs, and the prevalence of excessive polypharmacy increased by 22%, from 9.5% to 12% from 2006 to 2020. The use of three or more psychotropic drugs decreased by 13% (from 3.9% in year 2006 to 3.4% in year 2020). The use of “drugs that should be avoided in older adults unless specific reasons exist”, decreased by 59%, from 13% to 5.4%.

**TABLE 1:** Description of the study populations 2006–2020.

	2006	2007	2008	2009	2010	2011	2012	2013	2014	2015	2016	2017	2018	2019	2020
All, n	811,377	811,423	809,481	809,149	811,409	815,855	820,905	830,758	845,429	857,888	875,067	900,499	933,409	976,022	1,014,596
Age, mean	82.0	82.0	82.1	82.2	82.2	82.2	82.2	82.1	82.1	82.1	82.0	81.9	81.7	81.6	81.5
Females, %	60.8	60.6	60.4	60.2	59.9	59.6	59.3	58.9	58.5	58.1	57.7	57.2	56.8	56.3	56.0
Number of drugs, mean	4.4	4.4	4.5	4.4	4.7	4.8	4.6	4.6	4.7	4.8	4.8	4.7	4.6	4.6	4.7
Excessive polypharmacy															
Prevalence, %	9.5	9.3	10.1	9.6	10.9	11.0	9.9	10.1	10.7	11.2	11.4	11.0	10.5	11.0	11.6
Regional variation coefficient^a^, %	14.0	11.9	11.9	10.7	10.6	10.3	9.7	9.5	9.0	10.5	9.5	9.0	8.7	9.3	9.0
Use of 3 or more psychotropics															
Prevalence, %	3.9	3.7	3.8	3.3	3.9	4.0	3.6	3.5	3.8	4.0	3.8	3.4	3.1	3.3	3.4
Regional variation coefficient^a^, %	17.8	15.8	16.3	18.2	17.0	16.0	18.6	17.2	17.2	18.1	16.1	16.9	17.1	14.1	14.1
Drugs that should be avoided in older adults unless specific reasons exist															
Prevalence, %	13.1	12.2	11.9	10.7	11.0	10.6	9.0	8.2	7.6	7.2	6.7	6.3	5.8	5.5	5.4
Regional variation coefficient^a^, %	9.6	10.0	9.4	9.3	9.2	8.1	8.6	7.8	8.4	8.1	8.5	8.8	8.7	9.9	10.8

^a^ Standard deviation expressed as percent of the mean.

The coefficient of variation (CV) decreased from 14% in 2006 to 9% in 2020 for excessive polypharmacy and from 18% to 14% for the use of three or more psychotropic drugs. For “drugs that should be avoided in older adults unless specific reasons exist” the CV remained stable at around 10% during the study period.

**FIGURE 1:**
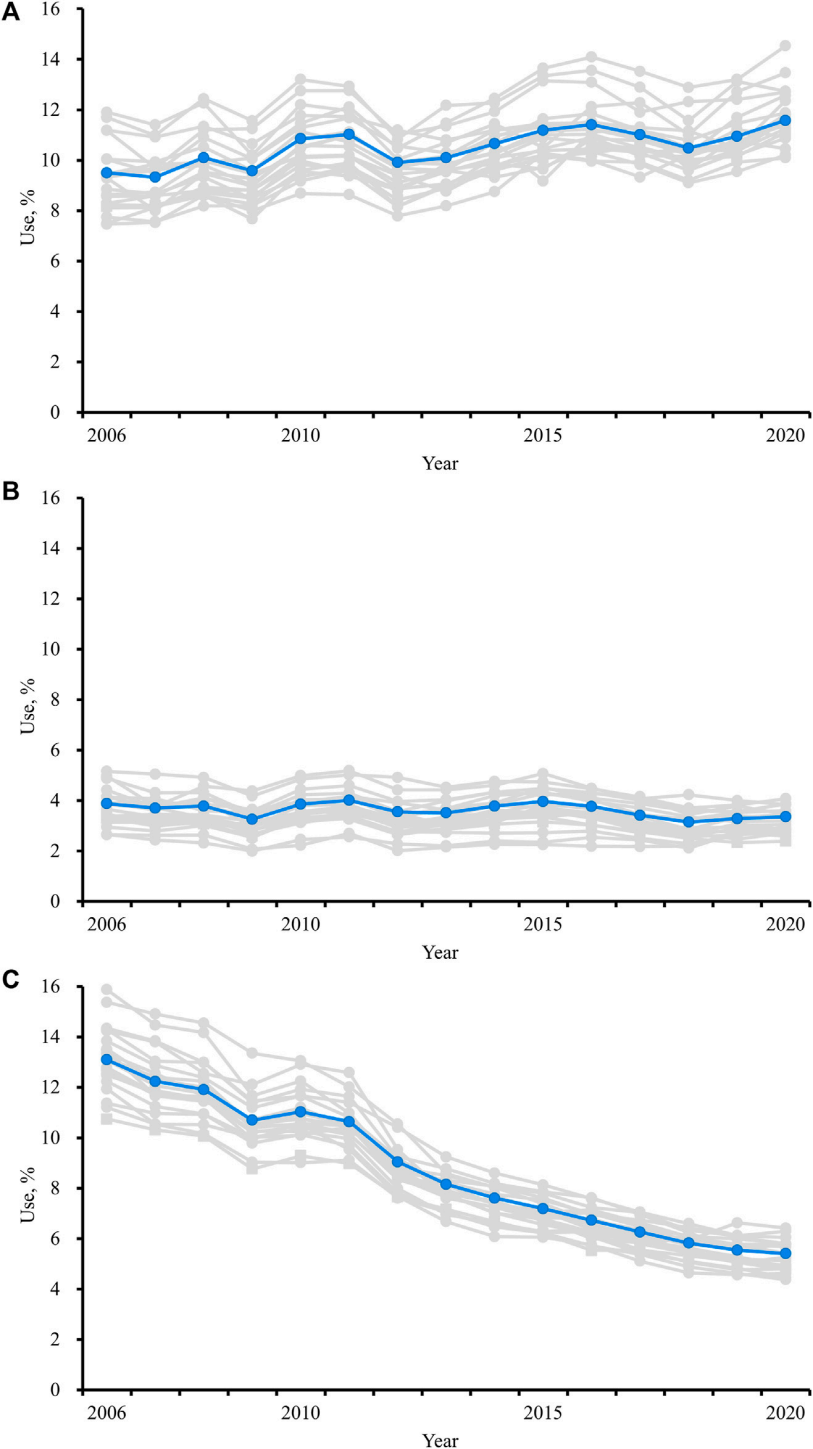
Prevalence of **(A)** use of 10 or more drugs, **(B)** use of three or more psychotropic drugs, **(C)** use of ‘drugs that should be avoided in older adults unless specific reasons exist’, in persons 75 years and older 2006–2020 in Sweden. Blue line: trend for the whole of Sweden. Grey lines: trends for the 21 different regions of Sweden.

The prevalence of excessive polypharmacy increased in all 21 regions from 2006 to 2020 (Figure 1A). The numbers supporting these figures is also reported in Supplementary Table S3A–C For the indicator “use of three or more psychotropics”, the prevalence decreased or remained stable in all but one region. (Figure 1B). Overall, zopiclone (ATC: N05CF01) was the most frequently used psychotropic drug in 2006 and 2020. The use of most of the specific psychotropic drugs declined during the period, with mirtazapine (ATC: N06AX11) as an exception (Supplementary Table S4). The prevalence of use of “drugs that should be avoided in older adults unless specific reasons exist” declined in all regions from 2006 to 2020 (Figure 1C). Of the drugs that should be avoided, all of the frequently used ones declined from 2006 to 2020, except a slight increase in the use of amitriptyline (ATC: N06AA09) which was the most prescribed inappropriate drug in 2020 (Supplementary Table S5).

**FIGURE 2:**
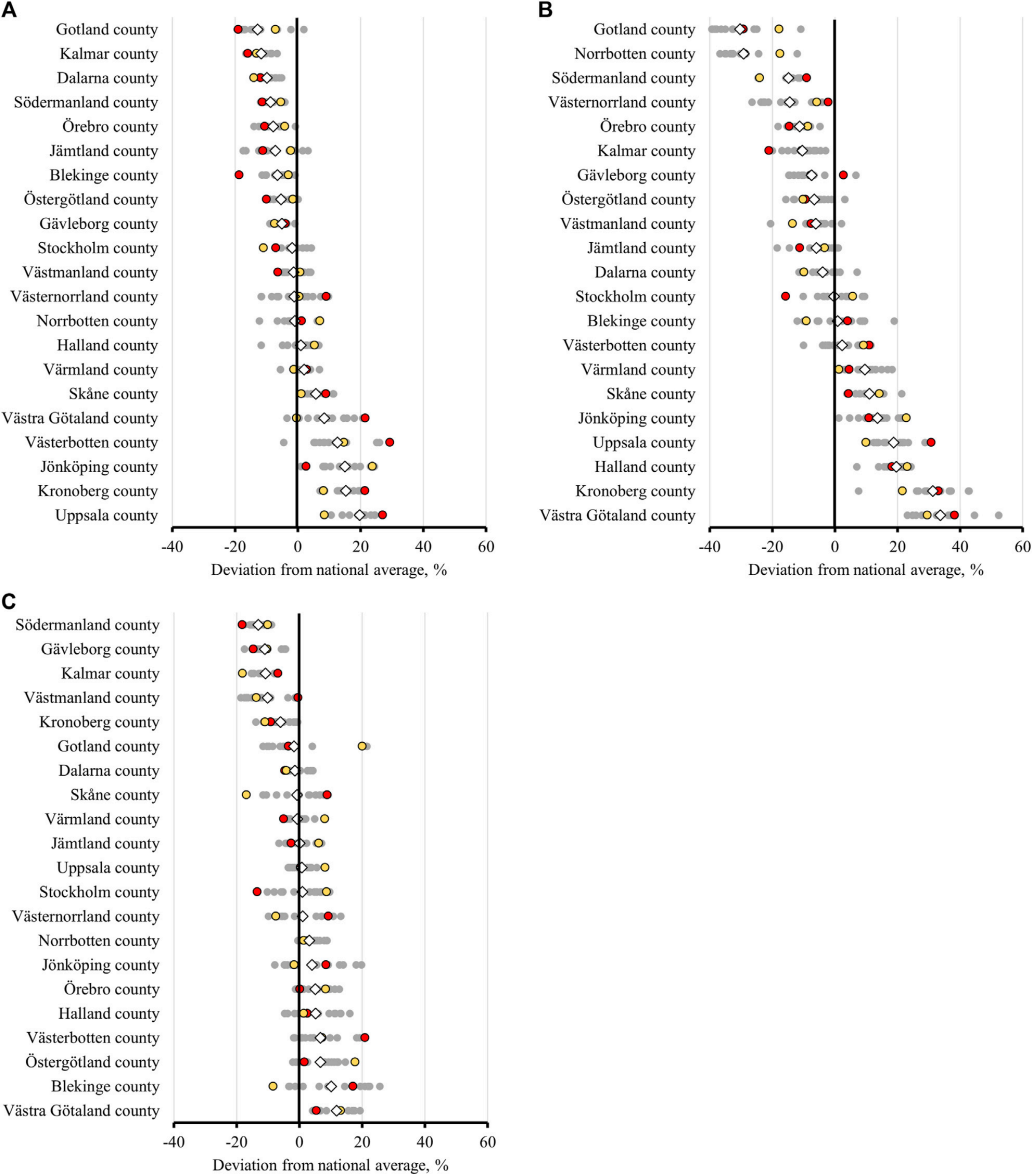
Prevalence of **(A)** use of 10 or more drugs, **(B)** use of three or more psychotropic drugs, **(C)** use of ‘drugs that should be avoided in older adults unless specific reasons exist’, in persons 75 years and older in the 21 regions of Sweden, 2006–2020. (⋄) average value for the years 2006–2020; (○) value for each year, red representing 2006 and yellow 2020.

In Figure 2 the deviation from the national average is presented, by region, across the study period, for each of the three indicators (Figure 2A–C). In each panel the regions are sorted by the mean deviation across the entire study period (depicted by the diamond). For each region, each year is represented by a dot, and the width of the horizontal dotted area indicates the total variation across time from the national average (the vertical zero-line). The red dot represents the first study year (2006) and the yellow dot the last study year (2020). Thus, the order of the red and yellow dot indicates the direction in which the regions are moving, closer or further away from the national average over time. In general, the pattern shows that some regions stay below or over the national average in all years. Moreover, with some exceptions, regions that deviate positively or negatively from the national average move closer to the mean across the period (i.e., the order of the yellow and red dot). The deviation from the national average is largest for the use of three or more psychotropic drugs (Figure 2B).

In a supplementary analysis, we depict the ranking of the regions across indicators to facilitate comparisons between regions in 2006 and 2020 (Supplementary Figure S1). In general, there is a pattern that regions performing in the top/bottom third on one indicator also are ranked in top/bottom third for the two other indicators.

## 4 Discussion

In this nationwide study of older adults aged 75 years and older from 2006 to 2020, we found a decline in the use of “drugs that should be avoided in older adults unless specific reasons exist” and the use of three or more psychotropic drugs, whilst the prevalence of excessive polypharmacy increased in all 21 Swedish regions. The regional variation decreased or was stable across the study period for all indicators, but was consistently largest for the “use of three or more psychotropic drugs”. We found a general pattern that regions with a good performance at the start of the period performed well across the entire period and *vice versa*. Moreover, regions performing well in one indicator was also more likely to perform well on other indicators. We regard the trends towards declining regional differences as positive since this increase the regional equality. Whether the remaining regional variations can be explained by contextual or individual/compositional factors needs to be investigated further.

We found that the prevalence of “drugs that should be avoided in older adults unless specific reasons exist” and the use of three or more psychotropic drugs declined from 2006 to 2020 in Sweden. The decline was especially evident for “drugs that should be avoided in older adults unless specific reasons exist” and was shared by all 21 Swedish regions. This decline is in line with previous research on trends in regional variation in drug use in older adults (Hogan et al., 2003), previous studies and reports from Sweden (Hovstadius et al., 2013; Thorell et al., 2020) and international studies of the trends in inappropriate medications (Stuart et al., 2003; Bongue et al., 2009; Lapi et al., 2009). This is likely explained by an overall increase in the awareness of which drugs to avoid in older adults. For the use of three or more psychotropic drugs the decline was, however, less pronounced, although only one region experienced an increase in this prevalence from 2006 to 2020. The moderate decrease for this indicator has also been reported previously in Sweden (Socialstyrelsen, 2016). In contrast, the prevalence of excessive polypharmacy increased in all 21 regions over the study period. The increasing prevalence of polypharmacy is also in line with previous results from Sweden and international studies (Wastesson et al., 2018). This can probably be inferred to the larger number of available drugs, an increased focus on diagnosing and treating chronic diseases and the increasing use of preventive medications. Overall, our results suggests that the Swedish regions tend to share a similar overall development for the studied indicators but regional differences in magnitudes exist.

We found that the differences between regions for the indicators decreased across the study period. The finding that the regional variation was smallest for ‘drugs that should be avoided in older adults unless specific reasons exist’ can possibly be explained by the fact that the strategies needed to avoid or substitute certain medications and medication classes, are relatively straightforward and therefore easier to implement. Thus, reducing the use of such drugs seems to represent a low hanging fruit compared to remedy other types of PID in older adults. The indicator “use of three or more psychotropic drugs” displayed the largest variation during the entire period. A high degree of variation between regions with regard to psychiatric polypharmacy have also been found in previous work (Okui and Park, 2022). Among potential explanation are regional prescribing patterns (e.g., opioid-belt in United States and benzo-belt in Sweden) or differences in access to specialist prescribers (Wastesson et al., 2014). Yet more detailed analyses of the drugs composing the indicator “use of three or more psychotropic drugs” in Sweden is needed.

Further, we found that the performance rankings between regions were relatively stable across time, similar to previous findings (Jirón et al., 2016). This stability, or path dependency, suggests that either contextual factors [e.g., therapeutic traditions (Ohlsson et al., 2009)] or individual/compositional factors (such as age structure) have been stable over the period (Morgan et al., 2010). The results of this study do not provide insights into the influence of these factors. Future studies in Sweden should attempt to study this in more detail. For example, the large cohorts born after 1945 will gradually join the age group of “persons 75 years and older”. This will result in a change in the age structure within this age group, resulting in a lowering of the mean age. This will potentially also result in a lowered prevalence of inappropriate drug use (in the situation that medication use is more appropriate in more recent cohort) if the age composition is not considered in analyses of “persons 75 years and older”. The importance of considering demographic changes in the composition of the old older adults will increase in the years to come as we are nearing a pivotal change in the age composition of this age group. In addition, regions who consistently performed well, or improved their rankings drastically during the period, could be more thoroughly examined, to identify successful strategies to reduce inappropriate drug use in older adults. This could potentially be done by mapping the Swedish regions’ strategies related to drug policy and incentives to promote rationale drug use over time (Eriksen et al., 2017).

The possibility to make a direct comparison between our results and other countries are somewhat limited. First, different criteria for PIDs are used in different countries and regions. This especially relates to “drugs that should be avoided in older adults unless specific reasons exist”, drugs considered inappropriate by one criterium can be considered appropriate according to other criteria. In order to partially circumvent this, we report the specific drugs most frequently contributing to the prevalence of “drugs that should be avoided in older adults unless specific reasons exist” in Sweden. We found that the most commonly used drug of that type in 2020 was amitriptyline which was used by about 1% of the study population. Amitriptyline is commonly reported as one of the most frequently used potentially inappropriate drug also in other countries and according to different criteria (Opondo et al., 2012). Second, international comparisons of psychotropic indicators are complicated due to differences in national drug formula across countries, for example no psycholeptics/psychoanaleptics combinations (ATC: N05C) are approved in Sweden. Last, we report a lower prevalence of excessive polypharmacy than most previous studies (Drusch et al., 2021). This is mainly explained by the use of a 1-day point prevalence in our study, that can be compared with the 3 and 12-month periods used in most other studies (Masnoon et al., 2017). Albeit, PID remains a problem in the old populations in most high-income countries, with 10%–20% affected (Tommelein et al., 2015). This likely results in adverse drug events, unnecessary hospitalisations and increased healthcare costs (Muhlack et al., 2017; Xing et al., 2019). Thus, it is of great importance to monitor trends and regional differences in potentially inappropriate drug use in different contexts. This can potentially help to identify successful strategies to reduce the level of PID.

### 4.1 Strengths and limitations

The main strength of this study is that the indicators were calculated using nationwide data with high validity from the Swedish Prescribed Drug Register (Wettermark et al., 2007). The study also has a number of limitations. Firstly, it only describes regional ecological trends in medication use in Sweden. We do not attempt to explain which factors contribute to the trends. Secondly, we decided to focus on three general and commonly used indicators of PID rather than all potential indicators of inappropriate drug use. Thirdly, drugs supplied in hospitals or nursing home storerooms are not recorded in the register, which could lead to an underestimation of inappropriate drug use. Fourthly, from the register data we know that the drug was dispensed but not whether it was consumed. In some cases, patients might have been informed to avoid drugs after it was dispensed, which would lead to an overestimation of PID use. Lastly, it should be noted that the National Board of Health and Welfare updated their set of indicators in 2017. In the present study, we use the 2010 version of the criteria to facilitate consistently measured indicators during the period.

## 5 Conclusion

This nationwide study shows that all Swedish regions shared a decline in the prevalence of “drugs that should be avoided in older adults unless specific reasons exist” and the use of three or more psychotropic drugs, whilst the prevalence of excessive polypharmacy increased, from 2006 to 2020. The regional differences decreased or were stable across the study period for all indicators. The differences were largest for the “use of three or more psychotropic drugs”. We found a pattern that regions with a good performance at the start of the period tended to perform well across the entire period and *vice versa*, with a few exceptions. In general, regional variations tended to be consistent across a 15-year period. More work is needed to identify the reasons for the regional variations. This could ultimately provide insights about strategies to improve quality of drug use in older adults.

## Data Availability

The data analyzed in this study is subject to the following licenses/restrictions: Individual data from the Swedish Prescribed Drug Register cannot be made publicly available. Requests to access these datasets should be directed to Registerservice@socialstyrelsen.se.
