# Supplementary material for: Regional variations in excessive polypharmacy and potentially inappropriate drug use among older adults in Sweden: Trends from 2006 to 2020
Source: Front Pharmacol. 2023 Feb 8;14:1030849. doi: 10.3389/fphar.2023.1030849 (PMC9945538; doi:10.3389/fphar.2023.1030849)
Supplement: Supplementary file 1 [file Table1.docx]

Supplementary Material

# Supplementary Table 1. Drug groups included in the indicator *Use of three or more psychotropic drugs*.

| **ATC-code** | **Drug group** |
| --- | --- |
| N05A | Antipsychotics |
| N05B | Anxiolytics |
| N05C | Hypnotics and sedatives |
| N06A | Antidepressants |

# Supplementary Table 2. Drug groups and drugs included in the indicator *Drugs that should be avoided in older adults unless specific reasons exist*.

| **ATC-code** | **Drug group / drug** |
| --- | --- |
| *Drugs with significant anticholinergic effects* | |
| A03AB | Drugs for functional gastrointestinal disorders, synthetic anticholinergics, quaternary ammonium compounds |
| A03BA | Drugs for functional gastrointestinal disorders, belladonna alkaloids, tertiary amines |
| A03BB | Drugs for functional gastrointestinal disorders, belladonna alkaloids, semisynthetic, quaternary ammonium compounds |
| A04AD | Other antiemetics |
| C01BA | Antiarrhythmics, class Ia |
| G04BD | Drugs for urinary frequency and incontinence - excl. G04BD12 (mirabegron) |
| N02AG | Opioids in combination with antispasmodics |
| N04A | Anti-Parkinson drugs, anticholinergic agents |
| N05AA | Phenothiazines with aliphatic side chain |
| N05AB04 | Prochlorperazine |
| N05AF03 | Chlorprothixene |
| N05AH02 | Clozapine |
| N05BB01 | Hydroxyzine |
| N06AA | Non-selective monoamine reuptake inhibitors |
| R05CA10 | Expectorants, combinations |
| R06AA02 | Diphenhydramine |
| R06AB | Antihistamines for systemic use, substituted alkylamines |
| R06AD | Antihistamines for systemic use, phenothiazine derivatives |
| R06AX02 | Cyproheptadine |
| *Long acting benzodiazepines* | |
| N05BA01 | Diazepam |
| N05CD02 | Nitrazepam |
| N05CD03 | Flunitrazepam |
| *Other* | |
| N02AX02 | Tramadol |
| N05CM06 | Propiomazine |

**Supplementary Table 3 (A-C).** Yearly prevalence of the three indicators by region 2006-2020 (presented in Figure 1).

# A. Use (%) of 10 or more drugs.

|  | **2006** | **2007** | **2008** | **2009** | **2010** | **2011** | **2012** | **2013** | **2014** | **2015** | **2016** | **2017** | **2018** | **2019** | **2020** |
| --- | --- | --- | --- | --- | --- | --- | --- | --- | --- | --- | --- | --- | --- | --- | --- |
| Stockholm county | 8.6 | 8.6 | 9.5 | 9.3 | 10.5 | 11.0 | 9.9 | 9.8 | 10.3 | 11.2 | 11.2 | 10.8 | 10.3 | 10.8 | 10.5 |
| Uppsala county | 11.7 | 11.0 | 12.4 | 11.6 | 13.2 | 12.9 | 10.8 | 12.2 | 12.3 | 13.3 | 13.6 | 12.9 | 11.6 | 13.1 | 12.7 |
| Södermanland county | 8.2 | 8.2 | 8.7 | 8.5 | 9.3 | 9.7 | 8.4 | 8.9 | 9.6 | 10.1 | 10.3 | 10.5 | 10.0 | 10.2 | 11.1 |
| Östergötland county | 8.3 | 8.6 | 8.8 | 8.6 | 9.8 | 9.8 | 8.8 | 9.8 | 10.3 | 11.0 | 11.0 | 10.5 | 9.8 | 10.4 | 11.6 |
| Jönköping county | 9.5 | 9.8 | 9.9 | 10.1 | 11.5 | 12.1 | 10.5 | 11.5 | 12.5 | 13.6 | 14.1 | 13.5 | 12.9 | 13.2 | 14.5 |
| Kronoberg county | 11.2 | 9.7 | 11.2 | 11.3 | 12.8 | 12.8 | 11.0 | 11.4 | 11.9 | 13.1 | 13.1 | 11.9 | 12.3 | 12.4 | 12.7 |
| Kalmar county | 7.7 | 7.6 | 8.2 | 8.2 | 9.2 | 9.6 | 8.9 | 9.1 | 9.7 | 10.1 | 10.3 | 9.8 | 9.1 | 9.5 | 10.2 |
| Gotland county | 7.5 | 7.5 | 8.6 | 8.0 | 8.7 | 8.6 | 7.8 | 8.2 | 8.7 | 10.3 | 10.0 | 9.3 | 10.2 | 11.2 | 10.9 |
| Blekinge county | 7.5 | 8.1 | 8.8 | 8.8 | 10.2 | 10.2 | 9.4 | 8.8 | 9.8 | 10.5 | 10.9 | 10.2 | 10.2 | 10.7 | 11.4 |
| Skåne county | 10.0 | 9.5 | 10.9 | 10.1 | 11.3 | 11.7 | 10.6 | 10.3 | 10.7 | 11.6 | 11.8 | 11.2 | 10.8 | 11.5 | 11.9 |
| Halland county | 9.3 | 8.0 | 9.5 | 9.0 | 10.5 | 11.0 | 9.9 | 10.2 | 11.2 | 11.4 | 11.5 | 11.3 | 11.2 | 10.5 | 12.4 |
| Västra Götaland county | 11.2 | 10.9 | 11.3 | 10.6 | 11.8 | 11.7 | 11.2 | 10.8 | 11.2 | 11.4 | 11.4 | 11.0 | 10.5 | 10.6 | 11.7 |
| Värmland county | 9.5 | 9.7 | 10.0 | 9.6 | 10.8 | 11.1 | 9.7 | 10.0 | 10.9 | 11.4 | 11.5 | 11.2 | 10.5 | 10.4 | 11.6 |
| Örebro county | 8.2 | 8.2 | 8.6 | 8.3 | 9.6 | 9.9 | 8.2 | 9.0 | 10.1 | 10.4 | 10.4 | 10.3 | 10.4 | 10.4 | 11.3 |
| Västmanland county | 8.6 | 8.7 | 10.0 | 9.5 | 10.2 | 10.2 | 9.1 | 9.6 | 11.0 | 11.4 | 11.4 | 10.8 | 10.4 | 10.5 | 11.8 |
| Dalarna county | 8.1 | 8.1 | 8.9 | 8.8 | 10.0 | 9.5 | 8.2 | 9.0 | 9.8 | 10.2 | 10.0 | 9.9 | 9.2 | 9.9 | 10.1 |
| Gävleborg county | 8.8 | 8.7 | 9.3 | 8.5 | 10.1 | 10.2 | 8.9 | 9.5 | 10.1 | 10.9 | 10.7 | 10.4 | 10.0 | 10.2 | 10.9 |
| Västernorrland county | 10.0 | 9.9 | 10.5 | 9.6 | 11.1 | 10.7 | 9.7 | 9.6 | 9.3 | 9.7 | 10.6 | 10.3 | 9.6 | 10.3 | 11.8 |
| Jämtland county | 8.2 | 8.2 | 8.6 | 7.7 | 9.6 | 9.4 | 8.8 | 9.6 | 10.7 | 9.2 | 11.1 | 11.3 | 9.6 | 10.9 | 11.5 |
| Västerbotten county | 11.9 | 11.4 | 12.3 | 10.2 | 12.2 | 12.0 | 10.0 | 10.7 | 11.4 | 10.5 | 12.1 | 12.3 | 11.0 | 12.7 | 13.5 |
| Norrbotten county | 9.3 | 9.6 | 9.8 | 9.0 | 10.6 | 10.5 | 9.4 | 9.6 | 9.9 | 9.6 | 10.8 | 10.8 | 10.2 | 11.7 | 12.6 |
| **Sweden, average** | **9.5** | **9.3** | **10.1** | **9.6** | **10.9** | **11.0** | **9.9** | **10.1** | **10.7** | **11.2** | **11.4** | **11.0** | **10.5** | **11.0** | **11.6** |

# B. Use (%) of three or more psychotropic drugs.

|  | **2006** | **2007** | **2008** | **2009** | **2010** | **2011** | **2012** | **2013** | **2014** | **2015** | **2016** | **2017** | **2018** | **2019** | **2020** |
| --- | --- | --- | --- | --- | --- | --- | --- | --- | --- | --- | --- | --- | --- | --- | --- |
| Stockholm county | 3.1 | 3.1 | 3.4 | 3.0 | 3.6 | 3.8 | 3.3 | 3.2 | 3.5 | 3.8 | 3.7 | 3.4 | 3.2 | 3.4 | 3.3 |
| Uppsala county | 4.9 | 4.3 | 4.3 | 3.5 | 4.2 | 4.3 | 3.6 | 4.0 | 4.3 | 4.2 | 4.3 | 4.1 | 3.4 | 3.8 | 3.5 |
| Södermanland county | 3.4 | 3.1 | 3.1 | 2.6 | 3.2 | 3.3 | 2.8 | 2.9 | 3.1 | 3.2 | 3.0 | 2.8 | 2.5 | 2.3 | 2.4 |
| Östergötland county | 3.4 | 3.5 | 3.4 | 3.2 | 3.5 | 3.6 | 2.9 | 3.1 | 3.4 | 3.6 | 3.3 | 2.8 | 2.6 | 2.6 | 2.8 |
| Jönköping county | 4.1 | 4.1 | 3.6 | 3.5 | 3.9 | 4.2 | 3.4 | 3.7 | 3.9 | 4.5 | 4.1 | 3.9 | 3.6 | 3.6 | 3.9 |
| Kronoberg county | 5.0 | 3.8 | 4.6 | 4.4 | 5.0 | 5.2 | 4.4 | 4.4 | 4.6 | 5.1 | 4.5 | 4.1 | 4.2 | 4.0 | 3.8 |
| Kalmar county | 2.9 | 2.8 | 3.0 | 2.5 | 3.3 | 3.5 | 3.1 | 3.1 | 3.2 | 3.5 | 3.4 | 3.0 | 2.6 | 2.9 | 2.8 |
| Gotland county | 2.6 | 2.6 | 2.6 | 2.1 | 2.2 | 2.7 | 2.0 | 2.2 | 2.3 | 2.3 | 2.2 | 2.2 | 2.2 | 2.8 | 2.6 |
| Blekinge county | 3.9 | 3.8 | 3.9 | 3.6 | 3.9 | 4.0 | 3.5 | 3.0 | 3.1 | 3.8 | 3.3 | 2.9 | 2.9 | 2.9 | 2.9 |
| Skåne county | 3.9 | 3.6 | 4.1 | 3.4 | 4.0 | 4.2 | 3.9 | 3.6 | 3.8 | 4.3 | 4.0 | 3.4 | 3.3 | 3.5 | 3.6 |
| Halland county | 4.4 | 3.7 | 4.1 | 3.6 | 4.4 | 4.6 | 4.0 | 4.0 | 4.3 | 4.5 | 4.3 | 4.0 | 3.7 | 3.5 | 3.9 |
| Västra Götaland county | 5.2 | 5.0 | 4.9 | 4.2 | 4.9 | 5.0 | 4.9 | 4.5 | 4.8 | 4.7 | 4.5 | 4.1 | 3.7 | 3.8 | 4.1 |
| Värmland county | 3.9 | 3.9 | 3.8 | 3.3 | 4.0 | 4.1 | 3.3 | 3.7 | 4.2 | 4.3 | 4.0 | 3.7 | 3.4 | 3.2 | 3.2 |
| Örebro county | 3.2 | 3.2 | 3.1 | 2.8 | 3.4 | 3.3 | 2.6 | 2.9 | 3.3 | 3.3 | 3.0 | 2.7 | 2.8 | 2.7 | 2.9 |
| Västmanland county | 3.4 | 3.2 | 3.4 | 2.8 | 3.5 | 3.8 | 3.1 | 3.2 | 3.6 | 3.5 | 3.5 | 3.1 | 2.7 | 2.5 | 2.7 |
| Dalarna county | 3.4 | 3.3 | 3.1 | 3.1 | 3.4 | 3.6 | 3.0 | 3.4 | 3.8 | 3.7 | 3.5 | 3.1 | 2.7 | 3.1 | 2.8 |
| Gävleborg county | 3.8 | 3.7 | 3.3 | 2.7 | 3.1 | 3.4 | 2.8 | 3.1 | 3.3 | 3.6 | 3.3 | 2.8 | 2.8 | 2.8 | 2.9 |
| Västernorrland county | 3.7 | 3.3 | 3.3 | 2.9 | 3.2 | 3.3 | 2.8 | 2.7 | 2.7 | 2.7 | 2.8 | 2.5 | 2.3 | 2.6 | 3.0 |
| Jämtland county | 3.3 | 3.0 | 3.2 | 2.5 | 3.6 | 3.5 | 3.0 | 3.3 | 3.5 | 3.5 | 3.6 | 3.2 | 2.8 | 3.0 | 3.0 |
| Västerbotten county | 4.1 | 3.7 | 4.0 | 3.0 | 3.8 | 3.8 | 3.2 | 3.3 | 3.5 | 3.3 | 3.5 | 3.4 | 2.9 | 3.3 | 3.4 |
| Norrbotten county | 2.7 | 2.4 | 2.3 | 2.0 | 2.5 | 2.6 | 2.3 | 2.2 | 2.4 | 2.3 | 2.5 | 2.4 | 2.1 | 2.7 | 2.6 |
| **Sweden, average** | **3.9** | **3.7** | **3.8** | **3.3** | **3.9** | **4.0** | **3.6** | **3.5** | **3.8** | **4.0** | **3.8** | **3.4** | **3.1** | **3.3** | **3.4** |

# C. Use (%) of ‘drugs that should be avoided in older adults unless specific reasons exist’.

|  | **2006** | **2007** | **2008** | **2009** | **2010** | **2011** | **2012** | **2013** | **2014** | **2015** | **2016** | **2017** | **2018** | **2019** | **2020** |
| --- | --- | --- | --- | --- | --- | --- | --- | --- | --- | --- | --- | --- | --- | --- | --- |
| Stockholm county | 11.4 | 11.0 | 10.9 | 10.0 | 10.4 | 10.3 | 9.0 | 8.2 | 7.8 | 7.5 | 7.0 | 6.5 | 6.1 | 6.0 | 5.8 |
| Uppsala county | 13.2 | 12.3 | 12.0 | 10.4 | 10.6 | 10.5 | 8.4 | 7.6 | 7.2 | 6.9 | 6.7 | 6.4 | 5.9 | 5.8 | 5.8 |
| Södermanland county | 10.7 | 10.3 | 10.1 | 8.8 | 9.3 | 9.0 | 7.7 | 7.2 | 6.7 | 6.2 | 5.5 | 5.6 | 5.1 | 4.8 | 4.8 |
| Östergötland county | 13.3 | 12.5 | 11.6 | 10.7 | 11.2 | 10.4 | 8.8 | 8.5 | 8.0 | 7.8 | 7.2 | 7.0 | 6.3 | 6.1 | 6.3 |
| Jönköping county | 14.2 | 13.8 | 12.5 | 12.1 | 12.9 | 12.6 | 9.5 | 7.8 | 7.0 | 6.8 | 6.3 | 6.1 | 5.5 | 5.0 | 5.3 |
| Kronoberg county | 11.9 | 10.5 | 10.5 | 10.0 | 10.1 | 10.4 | 8.6 | 7.6 | 7.2 | 6.7 | 6.0 | 5.8 | 5.6 | 5.1 | 4.8 |
| Kalmar county | 12.2 | 11.2 | 10.9 | 9.8 | 10.1 | 9.7 | 7.8 | 7.0 | 6.5 | 6.2 | 5.7 | 5.4 | 4.9 | 4.6 | 4.4 |
| Gotland county | 12.7 | 11.7 | 11.6 | 10.3 | 10.3 | 10.0 | 8.0 | 7.0 | 6.6 | 6.3 | 6.4 | 5.9 | 5.9 | 6.6 | 6.4 |
| Blekinge county | 15.4 | 14.9 | 14.5 | 13.3 | 13.1 | 12.0 | 10.5 | 8.6 | 8.0 | 7.4 | 6.6 | 5.9 | 5.6 | 5.3 | 4.9 |
| Skåne county | 14.3 | 13.0 | 13.0 | 11.4 | 11.7 | 10.8 | 9.2 | 8.1 | 7.4 | 6.7 | 6.1 | 5.4 | 5.1 | 4.8 | 4.4 |
| Halland county | 13.5 | 11.7 | 11.4 | 10.7 | 10.8 | 10.5 | 9.3 | 8.8 | 8.2 | 7.9 | 7.6 | 7.0 | 6.2 | 5.6 | 5.4 |
| Västra Götaland county | 13.8 | 12.9 | 12.4 | 11.2 | 11.7 | 11.4 | 10.4 | 9.2 | 8.6 | 8.1 | 7.6 | 7.1 | 6.6 | 6.1 | 6.0 |
| Värmland county | 12.5 | 11.9 | 11.5 | 10.2 | 10.5 | 10.1 | 8.5 | 7.9 | 7.4 | 7.1 | 6.4 | 6.2 | 6.0 | 5.6 | 5.8 |
| Örebro county | 13.1 | 12.4 | 12.2 | 10.5 | 11.0 | 10.5 | 8.7 | 8.3 | 8.1 | 7.6 | 6.9 | 6.7 | 6.4 | 6.1 | 5.8 |
| Västmanland county | 13.1 | 12.1 | 11.8 | 10.5 | 10.5 | 9.5 | 7.7 | 6.7 | 6.1 | 6.0 | 5.8 | 5.1 | 4.6 | 4.6 | 4.6 |
| Dalarna county | 12.5 | 11.8 | 11.5 | 10.5 | 10.7 | 10.4 | 8.4 | 8.1 | 7.7 | 7.1 | 6.5 | 6.2 | 5.4 | 5.2 | 5.1 |
| Gävleborg county | 11.2 | 10.5 | 10.2 | 9.0 | 9.0 | 9.1 | 7.6 | 7.1 | 6.5 | 6.2 | 6.3 | 5.8 | 5.4 | 5.1 | 4.8 |
| Västernorrland county | 14.3 | 13.8 | 13.0 | 11.4 | 11.9 | 11.6 | 9.2 | 7.7 | 7.0 | 6.5 | 6.2 | 5.5 | 5.3 | 5.1 | 4.9 |
| Jämtland county | 12.8 | 11.8 | 11.6 | 9.9 | 10.6 | 10.2 | 8.3 | 7.8 | 7.4 | 6.9 | 7.0 | 6.6 | 5.8 | 5.8 | 5.7 |
| Västerbotten county | 15.9 | 14.5 | 14.2 | 11.7 | 12.2 | 11.0 | 8.8 | 7.7 | 7.5 | 6.8 | 6.8 | 6.2 | 5.7 | 5.7 | 5.7 |
| Norrbotten county | 13.5 | 12.2 | 12.0 | 10.7 | 11.1 | 10.4 | 8.8 | 8.1 | 7.7 | 7.0 | 7.1 | 6.6 | 6.1 | 5.8 | 5.4 |
| **Sweden, average** | **13.1** | **12.2** | **11.9** | **10.7** | **11.0** | **10.6** | **9.0** | **8.2** | **7.6** | **7.2** | **6.7** | **6.3** | **5.8** | **5.5** | **5.4** |

**Supplementary table 4.** The 10 most frequently used psychotropic drugs, in 2006 and 2020.

|  | 2006 |  |  |  | 2020 |  |
| --- | --- | --- | --- | --- | --- | --- |
| ATC | Drug | Use, % |  | ATC | Drug | Use, % |
| N05CF01 | Zopiclone | 8.1 |  | N05CF01 | Zopiclone | 8.6 |
| N06AB04 | Citalopram | 6.6 |  | N06AX11 | Mirtazapine | 5.7 |
| N05BA04 | Oxazepam | 5.3 |  | N05BA04 | Oxazepam | 4.0 |
| N05CF02 | Zolpidem | 5.3 |  | N05CF02 | Zolpidem | 3.2 |
| N05CM06 | Propiomazine | 2.8 |  | N06AB04 | Citalopram | 2.7 |
| N06AX11 | Mirtazapine | 2.1 |  | N06AB06 | Sertraline | 2.5 |
| N05CD03 | Flunitrazepam | 1.6 |  | N06AB10 | Escitalopram | 1.2 |
| N05AX08 | Risperidone | 1.6 |  | N06AA09 | Amitriptyline | 1.2 |
| N06AB06 | Sertraline | 1.5 |  | N05CM06 | Propiomazine | 1.2 |
| N05BA01 | Diazepam | 1.2 |  | N05CH01 | Melatonin | 1.0 |

**Supplementary table 5.** The 10 most frequently used ‘drugs that should be avoided in older adults unless specific reasons exist’, in 2006 and 2020.

|  | 2006 |  |  |  | 2020 |  |
| --- | --- | --- | --- | --- | --- | --- |
| ATC | Drug | Use, % |  | ATC | Drug | Use, % |
| N02AX02 | Tramadol | 3.0 |  | N06AA09 | Amitriptyline | 1.2 |
| N05CM06 | Propiomazine | 2.8 |  | N05CM06 | Propiomazine | 1.2 |
| N05CD03 | Flunitrazepam | 1.6 |  | N05BB01 | Hydroxyzine | 0.6 |
| G04BD07 | Tolterodine | 1.5 |  | G04BD07 | Tolterodine | 0.5 |
| N05BA01 | Diazepam | 1.2 |  | N02AX02 | Tramadol | 0.5 |
| N05BB01 | Hydroxyzine | 1.2 |  | N05BA01 | Diazepam | 0.4 |
| N05CD02 | Nitrazepam | 0.9 |  | G04BD08 | Solifenacin | 0.4 |
| N06AA09 | Amitriptyline | 0.9 |  | R06AD02 | Promethazine | 0.3 |
| N05AA02 | Levomepromazine | 0.3 |  | R06AD01 | Alimemazine | 0.2 |
| G04BD08 | Solifenacin | 0.2 |  | G04BD11 | Fesoterodine | 0.1 |

**Supplementary figure 1.** Heat map of the rank order of Swedish regions regarding the use of 10 or more drugs, three or more psychotropic drugs and ‘drugs that should be avoided in older adults unless specific reasons exist’ (inappropriate drugs). 2020 compared to 2006. A lower rank equates a lower prevalence.

|  | Excessive polypharmacy | |  | Three or more psychotropics | |  | Drugs that should be avoided in older adults | |
| --- | --- | --- | --- | --- | --- | --- | --- | --- |
|  | 2006 | 2020 |  | 2006 | 2020 |  | 2006 | 2020 |
| Gotland county | 1 | 5 |  | 1 | 2 |  | 8 | 21 |
| Blekinge county | 2 | 8 |  | 13 | 8 |  | 20 | 7 |
| Kalmar county | 3 | 2 |  | 3 | 5 |  | 5 | 1 |
| Dalarna county | 4 | 1 |  | 7 | 7 |  | 7 | 9 |
| Södermanland county | 5 | 6 |  | 9 | 1 |  | 1 | 6 |
| Jämtland county | 6 | 9 |  | 6 | 12 |  | 9 | 13 |
| Örebro county | 7 | 7 |  | 5 | 9 |  | 11 | 17 |
| Östergötland county | 8 | 10 |  | 8 | 6 |  | 13 | 20 |
| Stockholm county | 9 | 3 |  | 4 | 14 |  | 3 | 18 |
| Västmanland county | 10 | 14 |  | 10 | 4 |  | 10 | 3 |
| Gävleborg county | 11 | 4 |  | 12 | 10 |  | 2 | 5 |
| Halland county | 12 | 16 |  | 18 | 20 |  | 14 | 11 |
| Norrbotten county | 13 | 17 |  | 2 | 3 |  | 15 | 12 |
| Jönköping county | 14 | 21 |  | 16 | 19 |  | 17 | 10 |
| Värmland county | 15 | 11 |  | 15 | 13 |  | 6 | 15 |
| Skåne county | 16 | 15 |  | 14 | 17 |  | 18 | 2 |
| Västernorrland county | 17 | 13 |  | 11 | 11 |  | 19 | 8 |
| Kronoberg county | 18 | 18 |  | 20 | 18 |  | 4 | 4 |
| Västra Götaland county | 19 | 12 |  | 21 | 21 |  | 16 | 19 |
| Uppsala county | 20 | 19 |  | 19 | 16 |  | 12 | 16 |
| Västerbotten county | 21 | 20 |  | 17 | 15 |  | 21 | 14 |

Sorted by lowest rank in Excessive polypharmacy in 2006
